# Gel Properties and Interaction Mechanism of Heat-Induced Lentinan–Chicken Myofibrillar Protein

**DOI:** 10.3390/foods14091614

**Published:** 2025-05-02

**Authors:** Kexin Li, Ya Ren, Yong Li, Liang Li, Yanfen Cheng, Shaojun Yun, Feier Cheng, Wenfei Zhao, Li Zhao, Mingchang Chang, Jinling Cao, Cuiping Feng

**Affiliations:** 1 College of Food Science and Engineering, Shanxi Agricultural University, Jinzhong 030801, China; 19722725980@163.com (K.L.); rybest0101@163.com (Y.R.); yong.li@sxau.edu.cn (Y.L.); scy_ll@163.com (L.L.); cyf2341986@163.com (Y.C.); yunshaojun@163.com (S.Y.); chengfeierm@163.com (F.C.); 18306891997@163.com (W.Z.); zhaoli19930405@163.com (L.Z.); sxndcmc@163.com (M.C.); 2 Shanxi Key Laboratory of Edible Fungi for Loess Plateau, Jinzhong 030801, China

**Keywords:** lentinan, chicken myofibrillar protein, gel properties, rheological properties

## Abstract

The enhancement of gel properties in chicken myofibrillar proteins (MPs) is a crucial objective in meat processing. In this experiment, we systematically investigated the effects of lentinan (LNT) on MP gel formation ability and three-dimensional network structure features through multi-scale structural characterization and molecular interactions analysis and elucidated the molecular pathways of their molecular actions in regulating gel properties. The addition of LNT (0–2%, *w*/*v*) significantly enhanced the water-holding capacity (WHC), textural, and rheological properties of LNT-MPs. As the concentration of LNT increased, the hydrophobic and electrostatic interactions became more pronounced. Conversely, the contribution from disulfide bonds exhibited an inverse relationship, with hydrogen bonds demonstrating the least impact. Subsequently, the α-helix content decreased from 23.75% to 22.64%, and the β-fold content increased from 28.03% to 29.22%, suggesting that the protein aggregates reorganized to form larger aggregates, which contributed to forming a more stable network structure of gels. This investigation establishes LNT’s capacity to modify the gelation mechanisms of MPs. These outcomes offer crucial insights for implementing fungal polysaccharides in processed meat product development.

## 1. Introduction

Myofibrillar proteins (MPs), serving as dominant structural proteins in meat systems, are of great significance in meat products and exert a profound influence on sensory, textural, emulsifying, and water-holding characteristics [1]. In processed meat manufacturing, the gelling process plays a pivotal role in achieving target product consistency through three-dimensional network formation [2]. Nevertheless, the viscoelastic properties of myofibrillar protein (MP) gels are relatively poor, and their applicability to specific textural attributes is restricted. This is primarily attributed to the low solubility of MP gels and the inadequate quantity of cystine necessary for the formation of disulfide bonds [3]. Consequently, enhancing the structural integrity of MP-based gels remains a priority in modern meat processing technologies.

Researchers have devoted considerable efforts to enhance the functionality of MP gels through exogenous substances such as polysaccharides and proteins through a variety of synergistic mechanisms, which has led to significant insights into the gelling ability of MPs [4]. Especially, the interactions between plant polysaccharides and proteins can form a ternary complex system of proteins, water, and polysaccharides with highly gelling properties [5]. Polysaccharides can optimize the performance of MP gels by optimizing the network structure, regulating the water distribution, enhancing the mechanical properties, and improving the protein structure [6]. For example, *tremella fuciformis* polysaccharide improves the rheological properties, texture, and thermal stability of the gel [7]. The addition of inulin, konjac glucomannan, and κ-carrageenan remarkably improves the water-holding capacity, textural characteristics, and rheological behavior of the gels [8]. Thus, the ability to improve the gel properties by incorporating polysaccharides into MPs is a potential strategy for enhancing the quality of meat products.

Lentinan (LNT), a polysaccharide extracted and isolated from the *Lentinus* edodes fruiting body, belongs to β-glucan and has been demonstrated to possess antioxidant [9], anti-tumor [10,11], anti-inflammatory [12], and immunomodulatory properties [13]. In proteoglycan systems, the major forces between polysaccharides and proteins have been shown to include hydrophobic interactions, electrostatic interactions, hydrogen bonding, and disulfide bonding [14]. In addition, the appropriate concentration of polysaccharides, which is one of the key factors influencing these forces, can enhance the stability of the gel network structure formed by MPs [15]. In the process of composite gel formation, the polysaccharide concentration has the most direct influence on the formation of the three-dimensional network structure. Only the optimal polysaccharide concentration can promote the incorporation of polysaccharides into the MP network, thereby contributing to the formation of a solid three-dimensional network structure. However, the effects and mechanisms of LNT concentration on MPs gel formation in chicken are not yet clear.

This study was designed to examine the impact of LNT addition (0–2%, *w*/*v*) on the characteristics of thermally induced LNT-MP gels, including water holding, oil holding, texture and conformation, particle size and zeta potential, solubility, and rheological properties. Furthermore, the interaction mechanism of LNT-MP gels at varying LNT concentrations was examined. This offers theoretical insights into the potential of LNT to enhance the quality characteristics of MP gel meat products.

## 2. Materials and Methods

### 2.1. Materials

Chicken breast meat used in this study was purchased from Jiajiali supermarkets in Taigu. The chicken breast meat used in this study was obtained from Sanhuang chickens (cocks) sold in the market at the age of 6–8 weeks, with a weight of 1600–2100 g.

All of the reagents, including KCl, MgCl_2_, Na_2_HPO_4_, NaCl, HCl, CuSO_4_, NaOH, potassium sodium tartrate, phosphate buffer PBS, SDS, Na_2_EDTA, and ammonium 1-anilino-8-naphthalenesulphonate, were purchased as analytically pure from Beijing Solarbio Science & Technology Co., Ltd. (Beijing, China).

### 2.2. Preparation of LNT

The enzyme-assisted extraction method was used to precisely put 100 g of *Lentinus edodes* powder into 4000 mL of distilled water. Five g of cellulase and neutral protease were added to hydrolyze *Lentinus edodes* powder for 3 h at 40 °C. Then, the mixtures were centrifuged at 7156× *g* and 25 °C for 20 min. The product was obtained by spin evaporation of the supernatant using a rotary evaporator (Model SENCOR220, Shen Sheng Technology Co., Ltd, Shanghai, China). Subsequently purified according to Wang’s method [16]. The prepared polysaccharide samples were more than 90% to meet the requirements of subsequent experiments.

### 2.3. MP Extraction

The MPs were extracted following the protocol described by Feng et al. [17] and subsequently stored at 4 °C. Configuration of protein extract: 7.455 g KCl, 0.4066 g MgCl_2_, and 3.5814 g Na_2_HPO_4_ were accurately weighed and mixed to a constant volume of 1000 mL. Then, the mixture was adjusted to a pH value of 7.0 with 20% HCl solution and stored in a reagent bottle. Configuration of protein eluent: 5.844 g of NaCl was weighed, the volume was set to 1000 mL, and it was stored in a reagent bottle.

Extraction of myofibrillar protein: fresh chicken breast was cleaned, fascia and fat were removed, and it was cut into small pieces. Seven g of processed chicken breast minced meat was put into a 100 mL centrifuge tube, 4 times the volume of protein extract was added, and it was homogenized at high speed for 30 s until fully mixed. Then, the mixture was freeze-centrifuged at 2 °C for 15 min at 2000× *g*. After centrifugation, the supernatant was discarded and the grease above the bottom was removed. This step was repeated three times to ensure that the extracted protein was pure. Next, 8 times the volume of protein eluent was added into the centrifuge tube, and high-speed homogenization and freeze centrifugation were performed at 2 °C for 15 min at 2500× *g* again. Similarly, the supernatant and oil were discarded after centrifugation; this step was repeated twice. Afterward, 8 times the volume of eluent was added again for homogenization and filtered with three layers of gauze to remove impurities. The pH value of the filtrate was adjusted to 6.2 using 0.1 M hydrochloric acid solution and mixed by magnetic stirring. After centrifugation, the supernatant was discarded to obtain pure myofibrillar protein. The extracted myofibrillar protein was transferred to a small beaker, and its absorbance was determined at a 540 nm wavelength. The MP concentration was determined using the Bradford method [18].

### 2.4. Preparation of LNT-MP Gels

We added a proper amount of MPs to LNT (*w*/*v*) solution containing 0%, 0.5%, 1%, 1.5%, and 2%, so that the final concentration of MPs was 60 mg/mL. Then, after mixing using a high-speed homogenizer (T18 brushless digital, IKA, Staufen, Germany), the composite gel was heated at 85 °C for 30 min and cooled to 25 °C to form a stable gel to be stored in a 4 °C refrigerator.

### 2.5. Determination of Water-Holding Capacity (WHC) and Oil-Holding Capacity (OHC)

The determination of WHC and OHC was based on the method of Wu et al. [19] with appropriate optimization. The LNT-MP hybrid gel and centrifuge tube were weighed (W_1_), then the samples were spun at 2795× *g* for 15 min to discard the supernatant (edible oil), and the precipitate and the centrifuge tube were weighed (W_2_). The WHC and OHC of the LNT-MP hybrid gel were computed using the following equations:

WHC (%) = (W_2_/W_1_) × 100%

OHC (%) = (W_2_/W_1_) × 100%

### 2.6. Determination of Rheological Characteristics

Viscoelasticity during gelation was determined using a MAL 1038384 rheometer (Malvern Panalytical Co., Ltd., Malvern, Worcestershire, UK). Subsequently, a linear viscoelastic region of 0.5% was determined for the LNT-MP samples, and frequency scans were performed within a frequency range of 0.1 Hz, recording the continuous variation of energy storage modulus (G′) and loss modulus (G″) values with shear rate [20].

### 2.7. Determination of Microstructure

The microstructure of LNT-MP hybrid gels was observed using a scanning electron microscope (Model SU 8010, Hitachi Corporation, Tokyo, Japan). Before analysis, the gels underwent freeze-drying and gold sputtering, and the observation was conducted at an accelerating voltage of 15 kV [21].

### 2.8. Determination of Texture

The LNT-MP composite gels were mechanically characterized by a professional texture analyzer (TA-XT plus, Stable Micro System, Godalming, Surrey, UK), and a P/50 cylindrical probe was chosen to perform the standard TPA detection mode. Each sample was cut into a 25 mm × 25 mm × 25 mm cube. The experimental parameters were calibrated as follows: The trigger threshold was 5 g, the pre-compression phase was carried out at a rate of 5.0 mm/s to approach the surface of the sample, the speed was reduced to 2.0 mm/s to perform a 10-mm fixed-pitch compression during the formal testing phase, and the probe returned to the initial position at 5.0 mm/s after the completion of the test [22]. All measurements were repeated three times at room temperature to take the average value.

### 2.9. Determination of Particle Size and Zeta Potential

Particle size and zeta potential of the LNT-MPs gels were measured by the instrument of British Malvern Instrument Co., Ltd, Malvern, UK. (Nano ZS90 Zeta potential particle sizer). Samples were diluted with PBS buffer to achieve the 1 mg/mL protein concentration [23].

### 2.10. Determination of Endogenous Fluorescence

Firstly, 10 mm phosphate buffer (PBS) with a pH value of 7.0 was prepared, and different LNT-MP gels were mixed. Samples were diluted with PBS buffer, and the dilution concentration was 0.1 mg/mL. Ten µL of 8.0 mm ANS (8-anilino-1-naphthalenesulfonic acid solution) with PBS buffer as solvent was added into 2 mL of the LNT-MP sample solution and reacted in the dark for 2 min. The endogenous fluorescence intensity of LNT-MPs (0.1 mg/mL) was performed by a G9800A fluorescence spectrophotometer (Agilent Ltd., Santa Clara, CA, USA) with the following parameters: excitation wavelength at 280 nm, emission wavelength ranging from 320 nm to 400 nm, and a slit width of 5 nm, as described by S. Zhao et al. [24].

### 2.11. Determination of Surface Hydrophobicity

The surface hydrophobicity of the LNT-MP composite gels was assessed according to the procedure reported by He et al. [25]. LNT-MPs were gradient diluted to different concentrations (0–0.02 mg/mL, 5 mL) using PBS buffer. Subsequently, they were mixed well with 1-anilinonaphthalene-8-sulfonic (ANS) (8 mmol/L, 10 µL) solution and placed in a dark environment at room temperature for 15 min. Surface hydrophobicity was quantified by measuring fluorescence intensity at 475 nm emission (with 280 nm excitation) and calculating the linear regression slope of the concentration-dependent fluorescence profile.

### 2.12. Determination of Free Sulfhydryl Content

The free sulfhydryl groups of LNT-MP composite gels (0%, 0.5%, 1%, 1.5%, and 2%) were determined using Ellman’s [26] method with minor modifications. Briefly, the sulfhydryl content was determined by reacting the MP suspension (1 mg/mL) with Tris-Gly buffer containing 4 mg/mL 5,5′-dithiobis (2-nitrobenzoic acid). The sample was incubated at 4 °C for 30 min, and the absorbance at 412 nm was measured. The sulfhydryl group concentration was calculated as follows:

SH (nmol/mg protein) = (73.53 × A_412_ × D)/C

where 73.53 = 106/(1.36 × 104), 1.36 × 104 is the molar extinction coefficient, D is the dilution factor, and C is the LNT-MP concentration.

### 2.13. Determination of Protein Solubility

LNT-MP samples were treated with four different buffer solutions to test their ability to break electrostatic interactions, hydrophobic interactions, hydrogen bonds, and disulfide bonds. A sample of LNT-MPs (2 g) was taken and placed into 10 mL of each of the four solvents: 0.05 M NaCl solution (S1), 0.6 M NaCl solution (S2), 0.6 M NaCl solution with 1.5 M urea (S3), and 0.6 M NaCl solution with 8 M urea (S4). Homogenization was carried out using a disperser (T18 brushless digital, IKA, Staufen, Germany) at 16,100× *g* for 2 min, followed by 30 min of standing at 4 °C. Finally, centrifugation was carried out at 4 °C for 15 min at 11,180× *g* using an Avanti J-26S XP centrifuge (Beckman Coulter, Brea, CA, USA) to obtain the supernatant, and the protein concentration was determined by the method of Lowry et al. [27]. The intermolecular interactions of MPs should be demonstrated by the solubility of the gel in different solutions.

### 2.14. Determination of Fourier Transform Infrared Spectroscopy (FTIR)

The LNT-MP composite gel lyophilized sample powder was mixed with potassium bromide in the ratio of 1:100 (*w*/*w*) in an onyx mortar, ground until a fine powder was obtained, and then pressed into tablets and tested by a Fourier transform infrared spectrometer (VERTEX 70, Bruker, Ettlingen, Germany) in the 4000–400 cm^−1^ range [28].

### 2.15. Determination of X-Ray Diffraction (XRD)

The X-ray diffractograms of the lyophilized LNT-MP composite gels were characterized at a rate of 2°/min with scattering angles ranging (2θ) from 5 to 60° at 25 °C using the X-ray diffractometer [29]. The diffraction angle was recorded with the corresponding diffraction intensity to generate the diffractograms. The experiment was repeated three times to obtain sufficient data points for subsequent analysis.

### 2.16. Statistical Analysis

The software GraphPad Prism 8 and Origin (2021) were used for data analysis and mapping. Each sample was repeated 3 times, and the average value was taken. The FTIR data were analyzed using OMNIC 7.3 and Peakfit v4.12 software. The Tukey test program in SPSS 27.0 was analyzed for significant difference (*p* < 0.05).

## 3. Results

### 3.1. Effects of LNT Addition on the Gel Properties of MPs

#### 3.1.1. WHC and OHC Analysis

**Figure 1:**
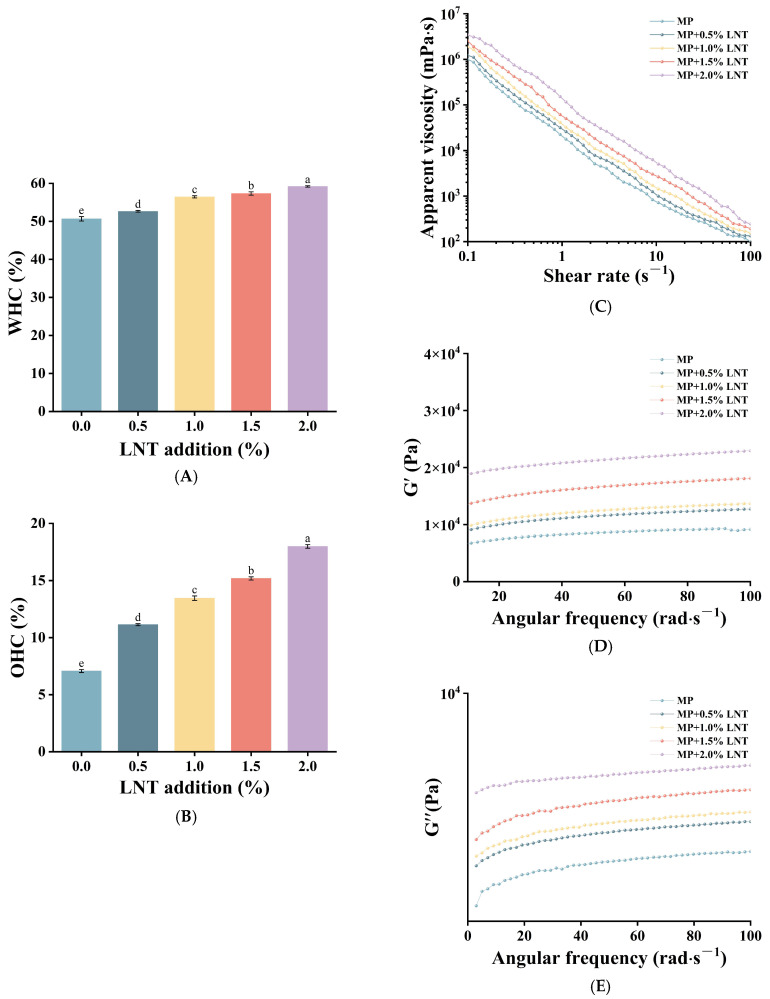
Water holding capacity (WHC) and oil holding capacity (OHC) properties and rheological characterization of chicken myofibrillar proteins (MPs) with added lentinan (LNT). Change in WHC (**A**); change in OHC (**B**); change in apparent viscosity (**C**); change in storage modulus (G′) (**D**); and change in loss modulus (G″) (**E**). Different letters (a–e) on top of a column indicate significant difference (*p* < 0.05) among samples treated under different lentinan additions.

WHC is an important measure of the performance of a gel network. Figure 1A shows the effect of adding LNT on gel WHC. After adding 2.0% LNT, the WHC of the composite gel increased to 59.19% compared with the control group (50.67%). This could be the fact that LNT has a synergistic effect with MPs, enabling the stable three-dimensional network structure to retain more water molecules. The study of Haijuan Nan et al. [30] also pointed out that the addition of LNT enhanced the WHC of the gel of chicken MPs. This may be due to the fact that LNT can be used as fillers and binders, and when added to the gel, they will fill into the network structure of the gel, making the gel structure more compact, thus improving the WHC capacity of the gel [31]. Concurrently, this network structure is able to lock up large amounts of water through physical constraints and hydrogen bonding. This finding is consistent with the article’s subsequent findings on electrostatic forces and hydrogen bonding.

As shown in Figure 1B, when the addition of LNT reached 2.0%, the OHC of the composite gel was significantly increased to 17.80% compared with the control group (7.08%). On the one hand, it is possible that the interaction between the LNT and MP molecules resulted in the partial defolding of the proteins, exposing some lipophilic groups that were previously masked inside the proteins, thus improving the OHC ability of the gels [32]. On the other hand, the interaction between LNT and MPs may induce intermolecular interactions that allow the edible oils to fill in sufficiently into the resulting continuous three-dimensional network structure, thus improving the OHC [33]. In addition, LNT molecules can adsorb on the surface of the oil droplets; with the increase of LNT, more oil droplets are encapsulated through spatial site-blocking effects and interfacial stabilization, thus enhancing the OHC of the gel [34].

#### 3.1.2. Rheological Analysis

Rheological analysis characterizes the viscoelastic nature of the gels and is an important indicator for assessing their structural properties and stability. Figure 1C showed the shear rate versus viscosity curves of the gels; with increasing shear rate, the apparent viscosity of each gel sample tended to decrease and eventually basically stabilized, which indicated that the gels had the pseudoplastic flow property of shear thinning [35]. Concomitantly, with the increase in LNT content, the apparent viscosity of the gel also rose significantly. This shows the addition of LNT promoted the degree of cross-linking between MPs and LNT molecules, which was helpful to improve the properties of the gel. It coincided with the study of Lizarraga et al. [36].

The effects of G′ and G″ on the angular frequency are illustrated in Figure 1D,E. The notable increase in G′ and G″ with rising LNT-MP concentration evinces augmented mechanical strength, which is corroborated by the findings of the texture analysis presented below. It is similarly conceivable that the expansion of LNT within the MP gels fills the mesh voids and promotes inter-particle connectivity, thereby facilitating the construction of a three-dimensional network structure. As illustrated in Figure 1D,E, the G′ values consistently exceed the G″ values, indicating a notable enhancement in the viscoelasticity and characteristics of the composite gels. This may be owing to the interactions between the MP and LNT molecules, which facilitate the formation of a more intricate and stable gel system.

#### 3.1.3. Scanning Electron Microscopy Analysis

**Figure 2:**
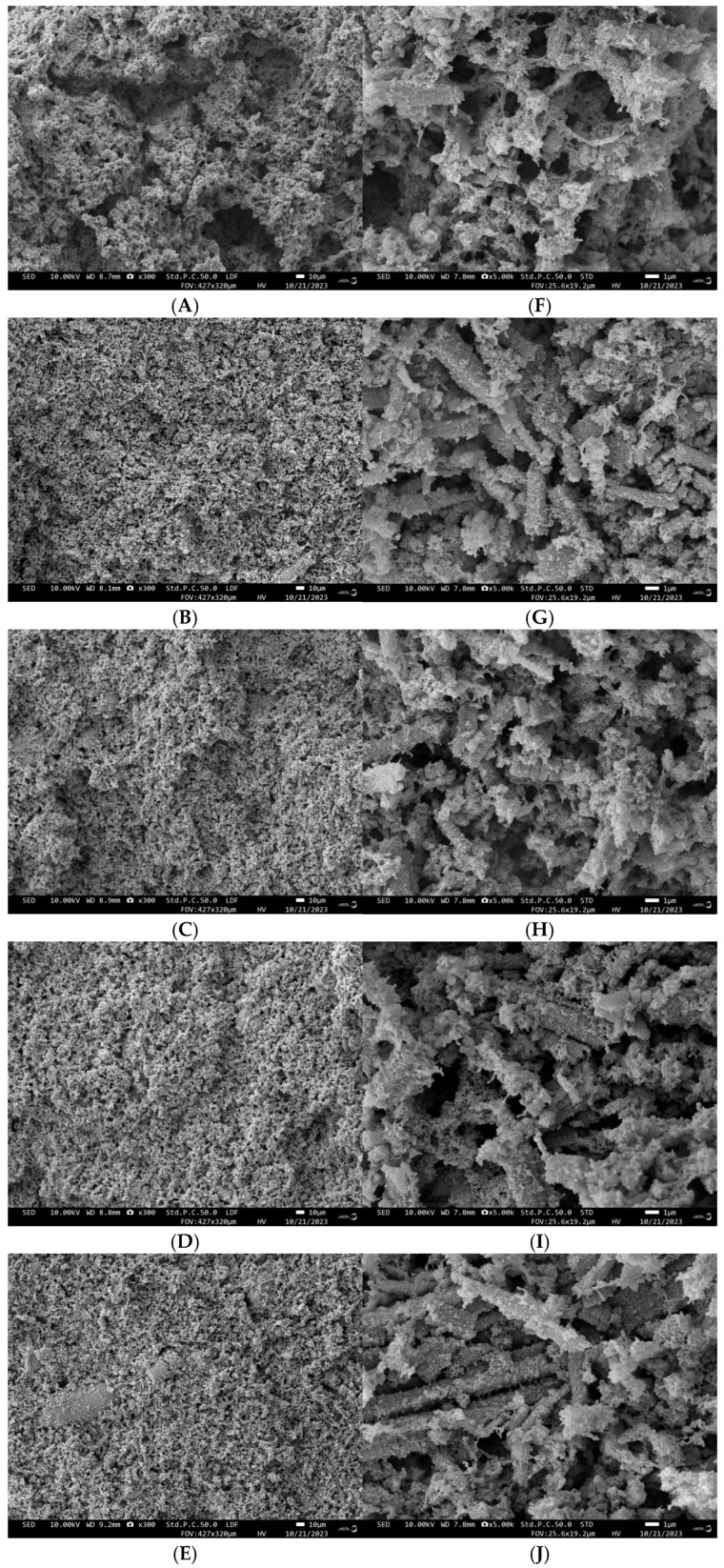
Scanning electron microscope of myofibrillar protein gel after adding different amounts of lentinan. (**A**–**E**) is the scanning electron microscope image of LNT-MP gels with 0%, 0.5%, 1%, 1.5%, and 2% LNT, respectively, at 300 times. (**F**–**J**) are the scanning electron microscope images of LNT-MP gels with 0%, 0.5%, 1%, 1.5%, and 2% LNT at 5000 times, respectively.

Figure 2 demonstrates the scanning electron microscope images (500× and 3000×) of the gels with different concentrations of LNT. The surfaces of all gel samples showed irregular pore structures with pores distributed over the entire surface. The gels without LNT possessed a rough network structure, while the gels with LNT exhibited a finer and tighter microstructure, which indicated an increased connectivity and tightness of the structure. The addition of 2% LNT resulted in a gradual decrease in pore size, accompanied by a tendency towards regularity, orderliness, and density. This led to an increase in WHC, potentially due to the enhanced binding capacity of the pores. This is consistent with the measured gel strength and fluorescence spectroscopy data, which indicate that the gels with LNT have higher gel strength and good cross-linking and compatibility. Q. Li [37] showed in the study that adding polysaccharide can make MP gel produce a more uniform and dense structure and improve gel strength and WHC.

#### 3.1.4. Texture Analysis

The textural properties of a food product are not only a key factor in assessing the sensory experience of the consumer but also a direct reflection of the product quality. The data presented in Table 1 clearly demonstrate that the incorporation of LNT had a pronounced impact on the textural properties of the gels, including cohesion, elasticity, viscoelasticity, chewability, and hardness (*p* < 0.05). The addition of LNT resulted in a notable increase in gel hardness (0.92 N) and elasticity (0.48 mm), which contributes to the formation of a rigid network structure. The findings indicate that the incorporation of LNT facilitates the molecular cross-linking of the composite gel system and enhances the stability of the MP binding, while a portion of the LNT serves as a filler within the gel, thereby improving the three-dimensional network structure. In summary, LNT is effective in enhancing the texture of the gel, especially at 2% LNT addition. Liu et al. [38] also reported that different additions of konjac polysaccharides significantly enhanced the hardness and springiness of the gel.

### 3.2. Effect of LNT Addition on the Interaction Mechanism of LNT-MP Composite Gels

#### 3.2.1. Particle Size and Zeta Potential Distribution Analysis

**Figure 3:**
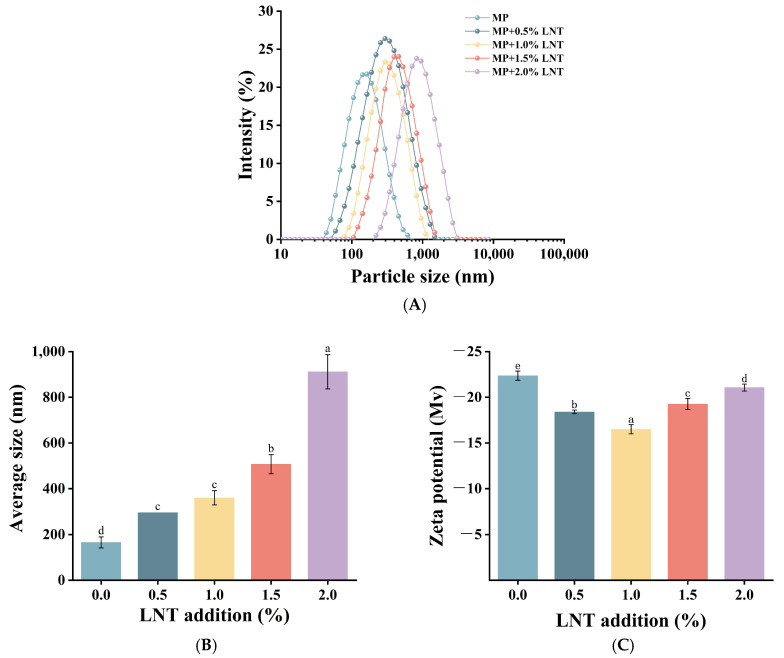
(**A**) shows the behavior of particle size distribution with LNT percentage, while the average particle size and zeta potential are shown in (**B**,**C**). Different letters (a–e) on top of a column indicate significant difference (*p* < 0.05) among samples treated under different lentinan additions.

As illustrated in Figure 3A, the incorporation of LNT markedly influenced the particle size distribution of the composite gels, with the distribution curve shifting towards larger particle sizes, which suggests that the formation of protein aggregates in the composite gel system is conducive to the development of dense and well-aggregated crosslinked structures of the composite gels [39]. The zeta potential serves as a metric for quantifying the intensity of intermolecular electrostatic interactions within gels [40]. As illustrated in Figure 3C, the gel exhibited a negative zeta potential, yet the potential of the gel demonstrated a tendency to decrease and then increase. This phenomenon may be due to the combination of the MP system and the LNT system, which increases the electrostatic repulsion within the gel system, leading to charge redistribution and a significant reduction in the gel’s electrical potential [41]. However, the augmented number of LNT forms more robust interactions with MPs, thereby forming a more compact three-dimensional network structure, which in turn results in an increase in zeta potential. Figure 3B demonstrates that the incorporation of LNT markedly (*p* < 0.05) augmented the mean particle size of the gel, indicating a greater adsorption of LNT on the MP surface. The multiple reactive groups in LNT facilitated its interaction with MP molecules, thereby contributing to the formation of a denser gel-stabilized structure.

#### 3.2.2. Endogenous Fluorescence Spectroscopy Analysis

**Figure 4:**
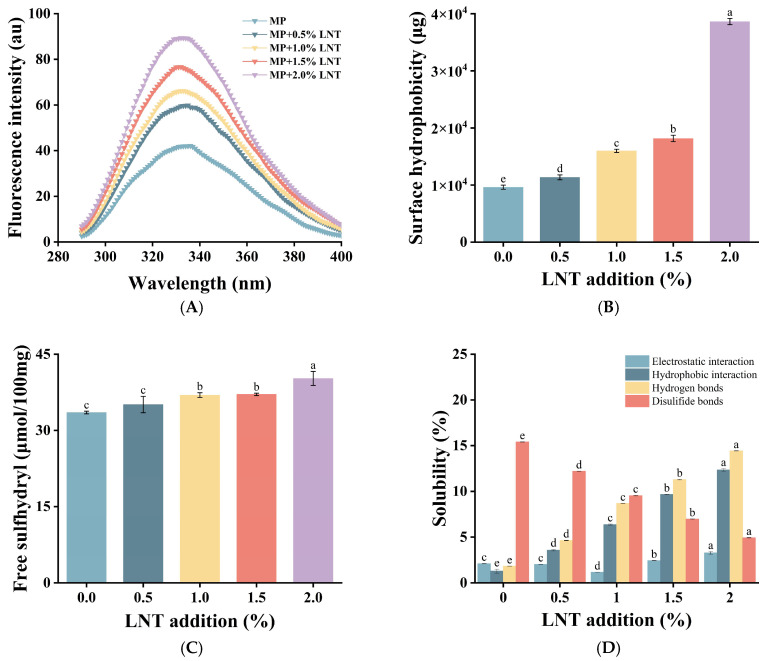
Changes in endogenous fluorescence (**A**), surface hydrophobicity (**B**), free sulfhydryl groups (**C**), and solubility (**D**) of chicken myofibrillar fibrillar proteins (MPs) with the addition of lentinan (LNT). Different letters (a–e) on top of a column indicate significant difference (*p* < 0.05) among samples treated under different lentinan additions.

Endogenous fluorescence spectroscopy is a way for assessing the tertiary structural changes of proteins by observing the fluorescence emission of tryptophan (Try) and tyrosine (Tyr) at an excitation wavelength of 290 nm. As shown in Figure 4A, the maximum fluorescence emission wavelength of the composite gel was red-shifted after the addition of LNT. This indicates that the incorporation of LNT alters the tertiary structure of MPs, exposing more tryptophan residues to a polar environment and thereby revealing hydrophobic regions and binding sites [42].

The change in fluorescence intensity was consistent with the maximum emission wavelength, suggesting that the interaction between LNT and MPs affects the sensitivity of tryptophan surroundings and, in turn, alters the three-dimensional conformation of the protein and the local microenvironment of fluorophores [43]. This change in the microenvironment may enhance the fluorescence output of fluorophores, leading to an enhancement of the endogenous fluorescence signal, which in turn affects the conformational unfolding of MPs and the properties of the gel [44].

#### 3.2.3. Surface Hydrophobicity Analysis

Surface hydrophobicity is a measure of the physicochemical properties of a protein surface [45]. Changes in surface hydrophobicity are owing to the expansion of the molecular structure of MPs, resulting in the disclosure of hydrophobic regions hidden within the proteins [46]. Figure 4B shows that the surface hydrophobicity of the gels increased significantly with the increasing LNT concentration (*p* < 0.05), peaking at 2% addition, which suggests that the addition of LNT strengthened the interaction with MPs, facilitated the exposure of the hydrophobic regions, and enhanced the surface hydrophobicity of the gels [47]. The observed enhancement of hydrophobicity is proved by the results of fluorescence detection. The increase of OHC also proves that the exposure of hydrophobic groups can promote the contact rate and affinity between protein and oil [48].

#### 3.2.4. Free Sulfhydryl Analysis

The free sulfhydryl level is usually used to indicate the state of disulfide bond formation in proteins [49]. As demonstrated in Figure 4C, the amount of free sulfhydryl groups within the composite gel was elevated by the addition of LNT. This may be due to the fact that the interaction of LNT with MPs induced the unfolding of MP molecules and changed their conformation [50], which in turn allowed the previously hidden sulfhydryl groups to be revealed and increased the amount of free sulfhydryl groups [51]. In addition, the functional groups, for instance hydroxyl and carboxyl groups, contained in LNT can interact with sulfhydryl groups in the amino acid residues of MPs to promote cross-linking with MPs, which releases part of the sulfhydryl groups from the original protein structure, reduces the formation of disulfide bonds, and results in an increased number of free sulfhydryl groups [52]. This observation is consistent with the results for disulfide bonds mentioned below.

#### 3.2.5. Solubility Analysis

The influence of LNT on the intermolecular forces of gels is presented in Figure 4D. The experimental results show that the main forces in gel formation are hydrophobic interactions and hydrogen bonding, while electrostatic interactions and disulfide bonding play a secondary role. As the concentration of LNT increased to 2%, the electrostatic interactions showed a pattern of first decreasing and then increasing, while the disulfide bonding interactions decreased by 10.5%, which were relatively small for these two types of forces. The interaction of LNT with MPs after heat treatment prompted a contact reaction between the two components, resulting in the stretching of the LNT molecular chains and the revelation of additional groups. By virtue of this process, the hydrophobic interactions were significantly boosted. As a direct consequence, there was a remarkable improvement in the structural stability as well as the functionality of the gels. The enhanced hydrophobic interactions were in accordance with the findings of the surface hydrophobicity. The observed increase in the number of hydrogen bonds may be ascribed to the hydroxyl and carboxyl groups of the LNT side chains, which are capable of forming robust hydrogen bonds with the amino and carboxyl groups of the MPs. Additionally, the self-crosslinking of hydrogen bonds in the LNT molecules may contribute to this phenomenon [53]. The reduction of disulfide bonds in the gel may be due to the interaction of hydroxyl groups on the LNT chains with sulfhydryl groups in MPs, thus protecting the sulfhydryl groups from oxidation to form disulfide bonds. This is consistent with the previous results of increased sulfhydryl groups.

#### 3.2.6. Fourier Transform Infrared Spectroscopy Analysis

**Figure 5:**
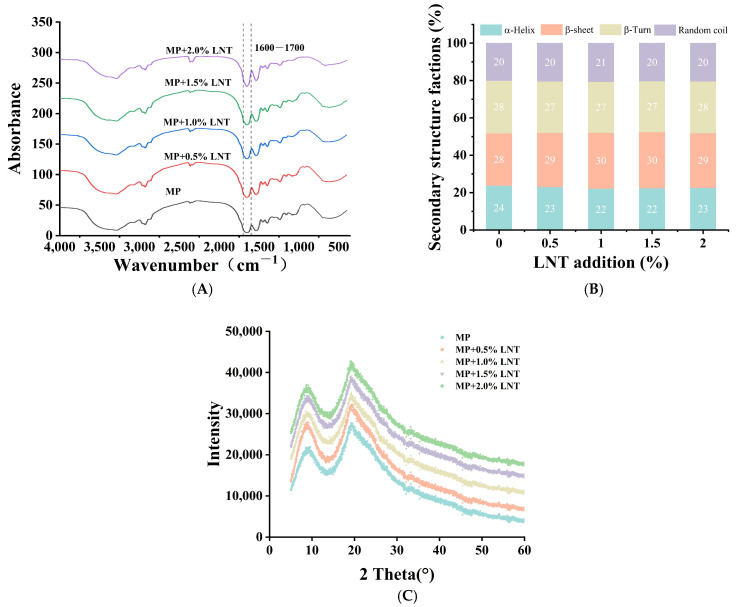
Fourier infrared spectra (**A**), changes in secondary structure content (**B**), and X-ray (**C**) diffraction patterns of chicken myofibrillar proteins (MPs) with added lentinan (LNT).

FTIR is a powerful tool for probing structural changes in protein gels at the molecular level [54]. The amide I region, illustrated in Figure 5A, lies in the wave number range of 1600 to 1700 cm^−1^ and is attributed to the stretching vibrations of the C-O and C-N bonds. The addition of LNT to the gels led to a notable broadening of the bands, indicating a modification in the protein structure during the gelation process. The quantitative results for each major secondary structure are presented in Figure 5B. The α-helical proportion of the gel significantly declined from 23.80% to 22.71%, while the β-folding content increased from 28.03% to 29.11%. Conversely, there was no conspicuous change detected in the β-sheet and irregular coil. The conformation of the MPs from the increase in the α-helical proportion and the decrease in the β-folded proportion indicated that a number of functional groups contributing to cross-linking between LNT and MP molecules were exposed. This is consistent with previous results on WHC and hydrophobicity.

#### 3.2.7. XRD Analysis

The molecular distribution in gel formation can be analyzed by observing the X-ray diffractograms, revealing details of the gel formation process and the aggregation structure [55]. Two sharp and high signal-to-noise diffraction peaks in the low-angle interval of 2θ < 30° were determined in the XRD patterns of gels with different LNT concentrations, which implies that LNT interacted with MPs to produce an amorphous complex or induce conformational recombination of MPs [56]. Figure 5C represents the XRD patterns of the gels formed with the addition of different concentrations of LNT; the addition of 0%, 0.5%, 1%, 1.5%, and 2% LNT all showed significant diffraction peaks at about 8.8 (weak) and 19.3 (strong), reflecting the short-range ordered or local interaction between molecules in the dynamic network of protein–polysaccharide complex. The molecular chains of MPs and LNT form local arrangements through hydrogen bonding or hydrophobic interaction. The broad peak indicates that this arrangement lacks periodicity, which is more likely to be a local order formed by the interaction between MPs and LNT in the gel network [57]. We found that the X-ray diffraction peak increased with increasing the percentage of LNT, which may be caused by the enhanced interaction between LNT and MP molecules during gel formation. Crystallinity also affects the WHC of gels, with higher crystallinity increasing the WHC of hydrogels. This is in agreement with the results of the previous WHC study. The intensity of the diffraction peak of the gel formed by incorporating 2.0% LNT is higher than that of other composite gels, indicating that a highly ordered semi-crystalline structure is formed between LNT and MP molecules. In addition, with the addition of LNT, the diffraction peak of the composite gel shifted red, indicating that the lattice row spacing of the gel decreased, which might be due to the interaction of LNT with MPs, resulting in a closer molecular arrangement. This is consistent with the previous results of increasing particle size.

## 4. Conclusions

This study demonstrates the efficacy of incorporating LNT into chicken MPs to improve its gel properties. As the concentration of LNT increased, a significant improvement was observed in the rheological and textural properties of the LNT-MP composite gels. Furthermore, 2% LNT creates a more efficient three-dimensional honeycomb network structure that promotes water absorption and enhances gel WHC. Additionally, LNT was able to fill the voids, thereby improving the interaction between the pores and consequently enhancing the apparent viscosity of the gels. Concurrently, the synergistic effect of hydrogen bonds, static electricity, and hydrophobicity in the gel can produce a more stable gel. The mechanochemical reinforcement was evidenced by the evolution of particle size, where progressive incorporation of LNT (0.5–2% *w*/*v*) induced electrostatic complexation between biopolymers, leading to controlled particle growth from 165.33 nm to 911.67 nm. LNT resulted in a transition between α-helix and β-folding and preserved the stability of the hydrogen bonding in the MPs, thereby promoting the gel developing a more refined and compact internal structural pattern. The formation of an ordered crystalline structure led to a more compact arrangement of molecules and the formation of smaller aggregates, which contributed to the stability of the gel. In conclusion, LNT, as an active ingredient of edible mushrooms, offers theoretical insight into the development of high-functional meat products and promotes the high-value utilization of edible mushroom resources in the food industry.

## Figures and Tables

**Table 1:** Effect of different lentinan content on texture of myofibrillar protein gel.

LNT Addition (%)	Cohesiveness (Ratio)	Springiness (mm)	Gumminess (N)	Chewiness (mJ)	Hardness (N)
0	0.21 ± 0.01 ^d^	1.56 ± 0.01 ^d^	0.43 ± 0.01 ^d^	0.69 ± 0.01 ^e^	1.83 ± 0.01 ^d^
0.5	0.22 ± 0.003 ^d^	1.74 ± 0.02 ^c^	0.50 ± 0.01 ^c^	0.91 ± 0.01 ^d^	1.87 ± 0.01 ^d^
1	0.24 ± 0.003 ^c^	1.75 ± 0.01 ^c^	0.57 ± 0.01 ^b^	1.02 ± 0.01 ^c^	1.93 ± 0.02 ^c^
1.5	0.26 ± 0.003 ^b^	1.85 ± 0.01 ^b^	0.59 ± 0.003 ^b^	1.06 ± 0.01 ^b^	2.60 ± 0.02 ^b^
2	0.31 ± 0.01 ^a^	2.03 ± 0.01 ^a^	0.62 ± 0.003 ^a^	1.15 ± 0.01 ^a^	2.75 ± 0.02 ^a^

Note: Different letters (a–e) on top of a column indicate significant difference (*p* < 0.05) among samples treated under different lentinan additions.

## Data Availability

The original contributions presented in the study are included in the article, further inquiries can be directed to the corresponding authors.
